# Interest to consider re-challenging by cetuximab and platinum containing regimen in recurrent Head and Neck Cancer

**DOI:** 10.18632/oncotarget.26506

**Published:** 2018-12-25

**Authors:** Christian Borel, Olivier Regnier-Gavier, Hélène Carinato, Sébastien Guihard, Delphine Antoni, Martin Demarchi, Florian Sirlin, Delphine Exinger, Emilie Petit-Jean, Alicia Thiery, Guy Bronner, Philippe Schultz, Henri Flesch, Véronique Frasie, Danielle Prébay, Thierry Petit, Alain C. Jung, Mickael Burgy, Pierre Coliat

**Affiliations:** ^1^ Medical Oncology Department, Centre Paul Strauss, Strasbourg, France; ^2^ Pharmacy Department, Centre Paul Strauss, Strasbourg, France; ^3^ Radiotherapy Department, Centre Paul Strauss, Strasbourg, France; ^4^ Supportive Care Department, Centre Paul Strauss, Strasbourg, France; ^5^ Biostatistics Department, Centre Paul Strauss, Strasbourg, France; ^6^ ENT Specialist, Strasbourg, France; ^7^ Université de Strasbourg, Inserm IRFAC UMR_S1113, group « STREINTH », Strasbourg, France; ^8^ Department of Otolaryngology Head and Neck Surgery, Hôpitaux Universitaires de Strasbourg, Strasbourg, France; ^9^ Radiobiology Laboratory, Centre Paul Strauss, Université de Strasbourg, Strasbourg, France; ^10^ Tumor biology Laboratory, Centre Paul Strauss, Université de Strasbourg, Strasbourg, France

**Keywords:** HNSCC, EXTREME, re-challenge, platinum free Interval

## Abstract

**Background:**

The EXTREME protocol is the standard of care for recurrent or metastatic head and neck squamous-cell carcinoma (R/M HNSCC) in first line. Beyond the first-line except immunotherapy, poor efficacy was reported by second-line chemotherapy. Re-challenge strategies based on a repetition of the first line with platinum and cetuximab regimens might have been an option to consider.

**Methods:**

We performed a retrospective study in order to assess the efficacy of the cetuximab plus platinum doublet-based chemotherapy regimen in patients with R/M HNSCC progressing after at least 3 months of cetuximab maintenance (EXTREME protocol). We complete a retrospective review of all medical records from R/M HNSCC patients treated after 16 weeks with the EXTREME regimen and treated with a re-challenge strategy between January 2010 and December 2014 in our institution (Centre Paul Strauss, Strasbourg, France).

**Results:**

33 patients were identified. The re-challenged strategy provided an ORR in 33.3% of cases and a DCR of 69.6% of cases. The median OS and PFS observed from the second line were 11.2 months and 6.5 months for the subset re-challenged by EXTREME or PCC regimens respectively. The response rate between patients with a platin free interval within 3 and 6 months and greater than 6 months were equal. Drugs dose intensity were better with the PCC protocol than the EXTREME regimen used as a rechallenge.

**Conclusions:**

This study suggest re-challenging strategy by these regimens could be considered beyond the first line as an option when the platin free interval is greater than 3 months.

## INTRODUCTION

The survival outcome of patients with a loco-regional or metastatic recurrences (R/M) of head and neck squamous carcinoma (HNSCC) is dismal. In 2008, the addition of cetuximab to conventional platinum/5FU chemotherapy (EXTREME regimen) has improved their overall survival [1]. These results have supported the EXTREME protocol approval as the standard of care for R/M HNSCC in first line.

Beyond the first-line when a progressive disease occurred, limited therapeutic options are available. Poor efficacy was reported by second-line chemotherapy [2, 3]. Of interest, immunotherapy including nivolumab or pembrolizumab, have provided encouraging results supporting their recent approval in 2nd line for R/M HNSCC [4, 5]. On the other hand, strategies based on a repetition of the first line with platinum and cetuximab regimens might have been an option to consider. Herein, we report a series of patients with R/M HNSCC, re-challenged in second line by a platinum and cetuximab-containing regimen.

## RESULTS

A total of 33 patients were re-challenged from 122 patients treated with the EXTREME protocol as the first line setting. Among them 18 and 15 were treated by PCC and EXTREME regimens respectively.

### Population characteristics

A total of 122 patients were treated in first line by EXTREME regimen. The OS and PFS observed for the 122 patients treated in first line setting were in line with the Vermorken study [1] (Data not shown). Among them, 33 patients eligible in the present study were re-challenged and their characteristics are summarized in Table 1. The majority were males (75.7%), the median age was 57 years. Tumour sites were predominantly oropharynx (42.4%) and larynx (21.1%). The OMS score was two for 36.4% (*n* = 12) of patients. Roughly half of patients had metastatic tumours (52%). All patients received an EXTREME protocol in first line and the carboplatin was the preferred drug (87.9%) compared with the cisplatin (12.1%). The first line with the EXTREME protocol achieved 22 OR and 11 SD. The PFI was within 3 and 6 months and longer than 6 months in 20 and 13 cases respectively.

**Table 1:** Population baseline characteristics

	*N* = 33 (%)
**Gender**	
Male	25 (76)
Female	8 (24)
**Age**	
Median	57
< 65 years	29 (88)
≥ 65 years	4 (12)
**Score OMS/Karnofsky**	
0–1	21 (64)
2	12 (36)
**Primary tumor localization**	
Oropharynx	14 (42)
Hypopharynx	6 (18)
Larynx	7 (21)
Oral cavity	6 (18)
**Tumor extension**	
Recurrent only	16 (48)
Metastatic disease	17 (52)
**Histologic type**	
Well differentiated	8 (24)
Moderately differentiated	11 (33)
Poorly differentiated	6 (18)
Missing	8 (24)
**1st Line extreme**	
Cisplatin	4 (12)
Carboplatin	29 (88)
**Response rate (1st line)**	*N* = 33
Complete Response	2 (6)
Partial Response	20 (61)
Stable Disease	11 (33)

### Primary objective

The re-challenged strategy provided an ORR in 33.3% [95% CI 17.2–49.4%] of cases and a DCR of 69.6% [95% CI 53.9–85.3%] of cases. A total, of 2 patients (6%) were not evaluable for response, 11 achieved a PR, 12 a SD and 8 a PD.

Among the 22 patients who experienced a response in first line, a second response in second line with EXTREME or PCC was reached in 8 patients (36.3%). Among the 11 patients with a SD in first line, an OR was achieved for 3 patients in second line with PCC (Table 2).

**Table 2:** ORR according to treatment (Extreme or PCC)

NB PTS	PR	SD	PD	NE	Total
**Extreme**	3 (20%)	7	4	1	15
	DCR = 66%				
**PCC**	8 (44%)	5	4	1	18
	DCR = 72%				
**Total**	11 (33%)	12	8	2	33

### Secondary objectives

**Figure 1:**
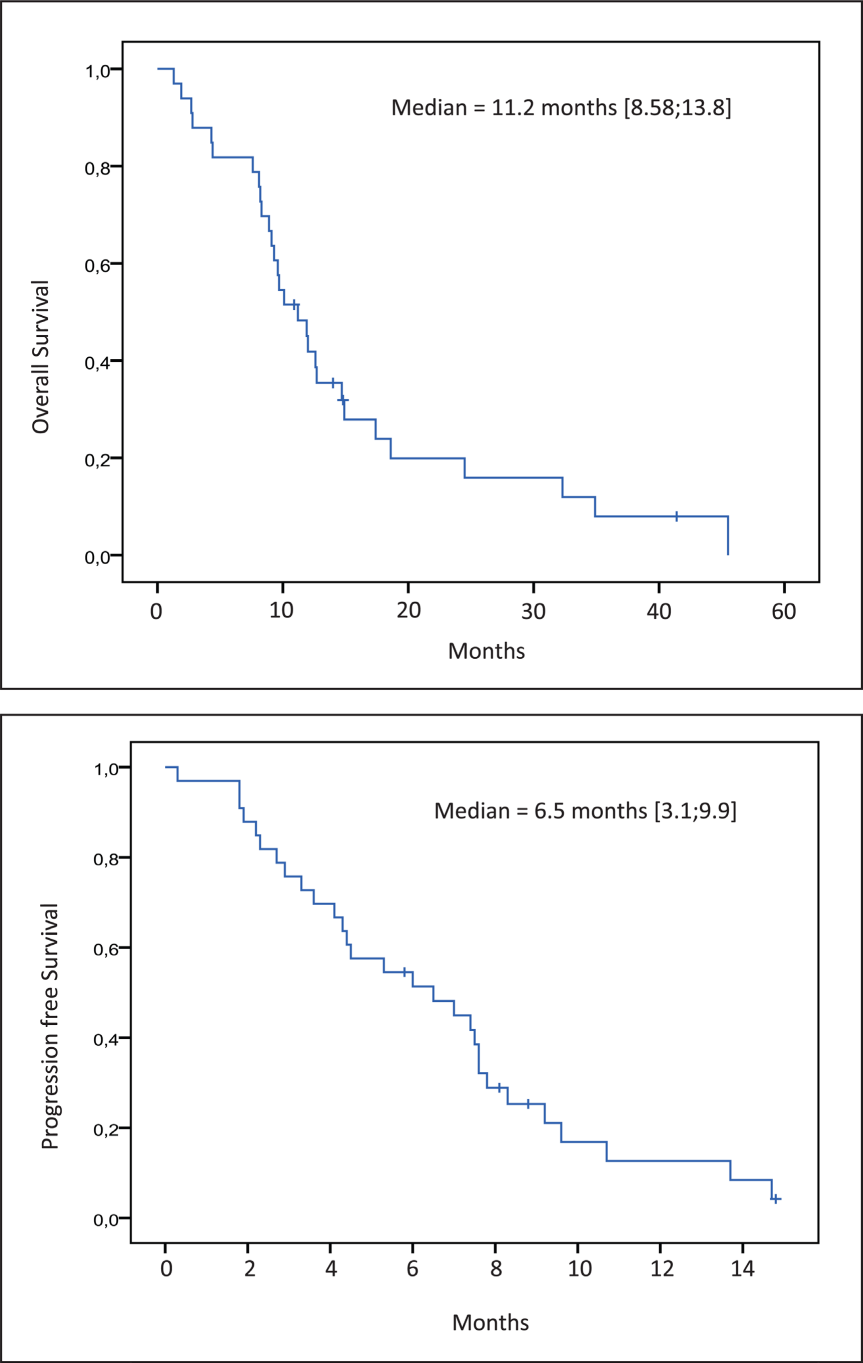
Kaplan-Meier estimate of overall survival and progression free survival

The survival, the median OS and PFS observed from the second line were 11.2 months (95% CI, 8.6–13.8) and 6.5 months (95% CI, 3.1–9.9) for the subset re-challenged by EXTREME or PCC regimens respectively (Figure 1).

The response rate between patients with a PFI within 3 and 6 months and greater than 6 months are statistically similar with an ORR of 30% (6/20) and 38% (5/13) and a DCR of 70% (14/20) and 69% (9/13) respectively (Table 3).

**Table 3:** Overall response rate according to PFI (Platinum free interval)

NB PTS	PR	SD	PD	NE
3 Mo < PFI < 6 Mo	6 (30%)^1^	8	4	2
	DCR = 70%^2^			
PFI > 6 Mois	5 (38%)^1^	4	4	0
	DCR = 69%^2^			
Total	11 (33%)	12	8	2
	DCR = 69%			

^1,2^: No statistical difference was observed for PR (Khi2 test: *p*-value = 1, CI 95:(0.19 5.7) OR = 1.02) and for DCR (Fischer exact test: *p*-value = 0.6894 CI 95 (0.2 10.7) OR = 1.53) between the 2 subsets (3 Mo < PFI < 6 Mo and PFI > 6 Mo)

Almost 20 weeks of treatment was achieved for cetuximab and paclitaxel, 15 weeks for carboplatin and 12 weeks for 5-fluorouracile. The median dose intensity for cetuximab, paclitaxel and 5-fluorouracile is between 80% and 90% of the planned dose per week (Table 4).

**Table 4:** Dose intensity

	Median total dose (mg/m^2^)	Median dose intensity (mg/m^2^/week)	Median total dose/median dose intensity (week)
**Cetuximab**	4016	211	19,0
**5-Fluorouracile**	14550	1210	12
**Paclitaxel**	1166	54	21.6
	**Median Total Dose (mg)**	**Median Dose Intensity (mg/week)**	**Median total dose/median dose intensity (week)**
**Carboplatin**	2450	165	14.8

Major toxicity of these protocols were neutropenia, anaemia and cutaneous toxicity. Side effects are in line with the drugs and protocols used and described in literature (Table 5).

**Table 5:** Toxicity

Toxicity	All grades (%)	Grades 3–4 (%)
**Neutropenia**	22 (64, 0)	8 (24, 0)
**Dysphagia**	9 (27)	7 (21)
**Cutaneous**	25 (76)	4 (12)
**Anemia**	25 (76)	3 (9)
**Thrombocytopenia**	17 (52)	2 (6)
**Neuropathy**	10 (30)	2 (6)
**Asthenia**	9 (27)	2 (6)
**Mucositis**	5 (15)	1 (3)
**Nausea, vomiting**	4 (12)	1 (3)
**Febrile neutropenia**	1 (3)	1 (3)
**Diarrhea**	1 (3)	1 (3)
**Thoracic pain**	3 (9)	-
**Infection**	4 (12)	-
**Hypersensitivity**	-	-

## DISCUSSION

In second-line treatment of R/M HNSCC, before the approval of immune checkpoint inhibitors, no standard has been defined: best supportive care or second line chemotherapies including monotherapy with methotrexate, paclitaxel, docetaxel or cetuximab were usually proposed [2, 6, 7]. In our study, among the 86 patients who could receive a second line chemotherapy, 53 were treated, according to these recommendations, with a single drug or a bi-therapy. The 33 others patients with a disease control of at least, 3 months after the 6 cycles of first line EXTREME chemotherapy where treated by a chemotherapy containing platinum and cetuximab with 5FU or paclitaxel. Interestingly, the observed efficacy results were quite similar to those observed in first line in terms of ORR, DCR and PFS.

The re-challenge strategy is an old concept and abundant example are present in the literature addressing several cancers [8]. The efficacy reported was supported by the tumour resistance concept. In the review of Kucynski et al., in 2013, several types of resistance were mentioned such as non-heritable drug resistance or a drug holiday-mediated tumour re-sensitization. The disease progression after the completion of a therapy was not necessarily related to drug resistance but only reflect the partial and temporary efficacy of the agent. The interval between the progressive disease occurrence might be an indicator of the possible tumour resistance regarding the previously given drugs. In R/M HNSCC a cytotoxic holiday over the cetuximab maintenance might be considered and our cohort appears qualified as a re-challenge approach.

In our present cohort, the sensitivity to platinum salts might be considered beyond an interval free of progression of 3 months. In the literature, it has been widely accepted that patients progressing within 6 months after the last platinum dose had a refractory disease. This is supported by 5 phases II studies performed in R/M patients who had progressed during or after platinum first line chemotherapy. Four of them were performed with a combination of cetuximab and a platinum salt which was reintroduced after the last 1st line chemotherapy administration [9–11]. Response rates reported in these clinical trials were ranged from 6% to 10%, and median survival were between 4.3 to 6.1 months. Interestingly, in the same early clinical recurrence, with a cetuximab monotherapy, a response rate of 13% and a 5,9 months median survival was reported [11]. Therefore, efficacy according to a PFI within 3 to 6 months and beyond 6 months was not investigated. In our study, similar results were observed between the two subsets with variable PFI. This point suggested that patients who progressed sooner than 6 months from the last platinum dose might not be refractory to the platinum salts in this setting.

## MATERIALS AND METHODS

### Patient’s selection

**Figure 2:**
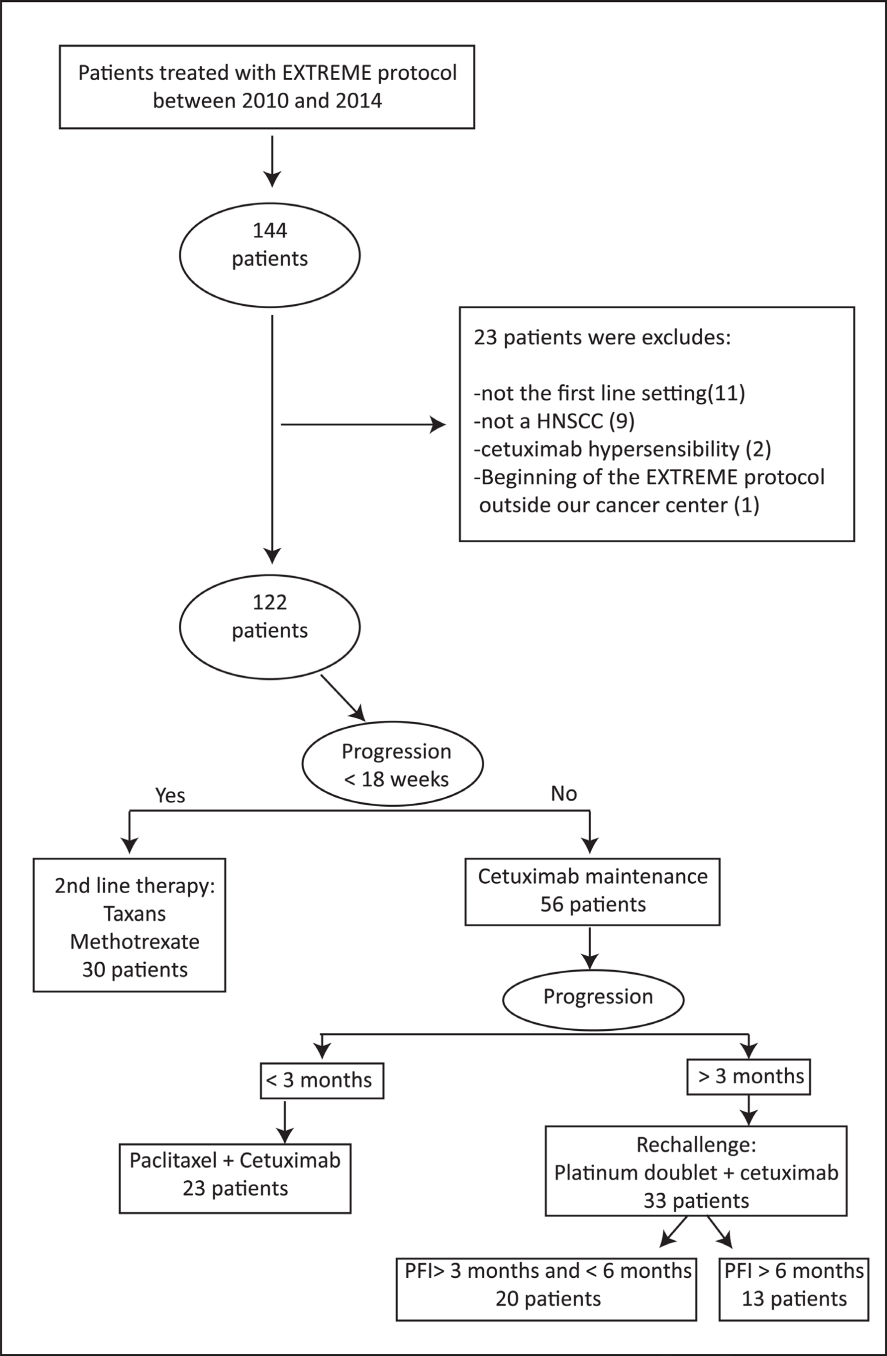
Selection process

From the computerized chemotherapy prescribing software database (AS400), all patients in the Paul Strauss Cancer Center from January 2010 and December 2014 treated in first line setting by the EXTREME regimen chemotherapy were identified. From the retrospective analysis of patient’s medical chart, treatment characteristics and outcomes were collected in compliance with the European data protection laws (RGPD). Patients with synchronous or second primary cancer, as well as patients with incomplete follow-up data were excluded. The common accepted strategy in our institution was to re-challenge with platinum and cetuximab regimen a patient with the occurrence of a progressive disease while they received cetuximab maintenance. The progression should occur beyond the third month after the completion of platinum from the EXTREME regimen. The selection process is explained in Figure 2. All follow-up data were updated until April 2016.

### Treatment

All eligible patients were treated in second line with the EXTREME protocol described by Vermorken et al. [1] or with the PCC protocol reported by Kies et al. [12]. EXTREME regimen included: cisplatin (100 mg/m^2^) or carboplatin (AUC 5) on day one (D1), 5-FU (1000 mg/m^2^ continuous infusion D1-D4) and cetuximab (loading dose (LD) of 400 mg/m^2^ followed by 250 mg/m^2^ infusions (D1, D8, D15)). Administration of chemotherapy was repeated every 3 weeks (D1 = D22) with a maximum of 6-cycles, followed by maintenance administration of cetuximab given weekly (250 mg/m^2^) or bi-weekly (500 mg/m^2^). PCC regimen included: Weekly Paclitaxel 80 mg/m^2^ + Carboplatin AUC2 + Cetuximab 250 mg/m^2^ (LD = 400 mg/m^2^) on D1 with a maximum of 16 courses, followed by maintenance administration of cetuximab given weekly or bi-weekly.

### End-points

This study was aimed to analyse the efficacy of re-challenge strategy in a 2nd line setting, in recurrent or metastatic Head and Neck Cancer with cetuximab plus platinum regimen in real life population.

The primary end-point was to evaluate the response rate in patients re-challenged. The response rate is composed with 2 criteria: The Overall response Rate (ORR) (Complete Response (CR) + Partial Response (PR)) and The Disease Control Rate (DCR) (ORR + Stable Disease (SD)). Responses were defined according to the RECIST criteria.

Secondary end-points were second line Overall Survival (OS: time from the onset of 2nd line chemotherapy to death or to last follow-up) and 2nd line Progression Free Survival (PFS: time from the onset of 2nd line chemotherapy to death or progression).

The relationship between the platinum free interval (PFI) (within 3 and 6 months or longer than 6 months) and endpoints was investigated.

Toxicity and dose intensity data were also collected.

### Statistical analysis

The statistics were mainly descriptive. OS and PFS were estimated with the Kaplan-Meier method. The response rate (OOR and DCR) comparison between the PFI subsets (3–6 months and > 6 months) was performed by Khi2 test or Fischer exact test. Statistical calculations were performed with SPSS^®^ v.22 (IBM^®^).

## CONCLUSIONS

In conclusion, at progression, treatment-containing platinum based chemotherapy and cetuximab (EXTREME or PCC chemotherapy) provided interesting results. Re-challenge by these regimens could be considered beyond the first line as an option when the PFI is greater than 3 months.
